# Development and Characterization of Polylactic Acid-Resveratrol-Based Polymeric Nanoparticles and Their Cytotoxic Effect on Two Breast Cancer Cell Lines in Combination with Doxorubicin

**DOI:** 10.3390/pharmaceutics18080974

**Published:** 2026-08-08

**Authors:** Laura Denise López Barrera, Kevin Yair Santillan Morales, Joselo Ramón Martínez Rosas, Elizabeth Soria-Castro, Patricia Ramírez Noguera, Flora Adriana Ganem Rondero, Roberto Diaz-Torres

**Affiliations:** 1 Laboratorio 9, Toxicología Celular, UIM, Facultad de Estudios Superiores Cuautitlán, Universidad Nacional Autónoma de México, Km 2.5 Carretera Cuautitlán-Teoloyucan, San Sebastián Xhala, Cuautitlán Izcalli 54714, Estado de México, Mexico; laura.lobar@cuautitlan.unam.mx (L.D.L.B.); 318314253@cuautitlan.unam.mx (K.Y.S.M.); mvzjoselomartinez@gmail.com (J.R.M.R.); ramireznoguera@unam.mx (P.R.N.); 2 Department of Cardiovascular Biomedicine, National Institute of Cardiology Ignacio Chávez, Juan Badiano No. 1, Col. Sección XVI, Tlalpan, Mexico City 14080, Mexico; sorieli23@yahoo.com; 3 Laboratorio de Investigación y Posgrado en Tecnología Farmacéutica (L-322, Campo 1), Facultad de Estudios Superiores Cuautitlán, Universidad Nacional Autónoma de México, Cuautitlán Izcalli 54740, Estado de México, Mexico; ganemq@hotmail.com

**Keywords:** nanoparticles, resveratrol, antioxidant, breast cancer

## Abstract

**Background/Objectives:** Resveratrol (RSV) is a phenolic compound that possesses antioxidant properties and whose biological application has been limited by its physicochemical properties, including its low solubility and stability. **Methods:** In this study, polymeric nanoparticles (NPs) were developed using polylactic acid (PLA) to encapsulate resveratrol and improve its bioavailability, using a factorial design to optimize the formulation and evaluate its cytotoxicity against breast cancer cells. **Results:** The optimized system presented an average size of 389.2 nm, a PDI of 0.127, a negative z-potential, and an encapsulation efficiency of 76.02%. The statistical analysis indicated that the PLA: RSV ratio was the main determinant of encapsulation percentage and particle size (*p* < 0.05). On the other hand, with the hemolysis test, blood compatibility was evidenced at all concentrations evaluated, while in the cell assays, the PLA-RSV nanoparticles significantly decreased the cellular viability of MCF-7 and MDA-MB 231 cells compared to untreated cells, and in combination with NP PLA-RSV and doxorubicin, greater cytotoxicity was produced in MCF-7 cells compared to nanoparticle-only treatments (*p* < 0.05, ANOVA, via followed by a Fisher test). **Conclusions:** In addition, distinct cytotoxic effects are observed, associated with their tumor phenotype. Taken together, these results suggest that PLA-RSV NPs are a useful system for future studies on redox status and other biological processes important in cancer.

## 1. Introduction

Breast cancer continues to be one of the leading causes of death worldwide, which is why therapeutic strategies that are more effective and safer to improve tumor selectivity and therefore reduce the adverse effects of conventional treatments continue to be developed [1]. In this context, nanotechnology offers an alternative by developing nanoformulations of natural active ingredients that serve as therapeutic agents, thereby reducing limitations of conventional drugs such as low solubility, rapid metabolic degradation, and limited bioavailability [2,3].

Considering this, resveratrol (3,5,4′-trihydroxystilbene), a plant-derived polyphenol, has been shown to exhibit anticancer, antioxidant, and anti-inflammatory properties in preclinical studies across various cancer types, including breast cancer. This molecule modulates signaling pathways associated with cell proliferation, apoptosis, and oxidative stress [4,5]. However, its clinical application is limited due to its physicochemical properties, including low aqueous solubility, limited chemical stability, and rapid metabolism, which compromise its therapeutic efficacy [3].

The nanoencapsulation of resveratrol using biodegradable polymers is a strategy proposed to overcome its physicochemical limitations and thereby increase intracellular uptake in tumor cells compared to free resveratrol [2]. Nanoparticles derived from polymers such as polylactic acid (PLA) may offer advantages, including improved biocompatibility, biodegradability, and a controlled degradation profile, positioning them as an alternative for drug delivery in oncology [6]. However, despite growing interest in the nanotoxicology of this type of PLA-resveratrol system, it has not yet been fully characterized, especially in breast cancer models. This approach will provide key information to develop nanostructured systems for future preclinical and clinical studies.

In this context, the present research aims to develop and optimize resveratrol-loaded PLA nanoparticles, evaluate their cytotoxicity in two breast cancer cell lines, and establish relationships between physicochemical properties and biological efficacy.

## 2. Materials and Methods

### 2.1. Chemicals

Polylactic acid (PLA) (Sigma-Aldrich, Toluca, Mexico, 38534), acetone (Sigma-Aldrich, Toluca Mexico, W332607), Polyvinyl alcohol (Sigma-Aldrich, Toluca, Mexico, 81383), and resveratrol (Merck, Darmstadt, Germany, 554325). For cell culture, the cell lines MCF-7 (ATCC HTB-22 cell line, official name MCF7, RRID: CVCL_0031, obtained from ATCC (Manassas, VA, USA), 2017) and MDA-MB-231 (ATCC HTB-26 cell line, official name MDA-MB-231, RRID:CVCL_0062, obtained from ATCC (Manassas, VA, USA), 2017) were used. DMEM/F12 + Gluta MAX TM (1×) (Gibco, Brooklyn, NY, USA), Fetal Bovine Serum (Gibco, Brooklyn, NY, USA), Penicillin/Streptomycin (Gibco, Brooklyn, NY, USA), Trypsin (10×) (Gibco, Brooklyn, NY, USA). To evaluate cytotoxicity, the following were used: Resazurin and Crystal Violet (Sigma-Aldrich, San Louis, MO, USA). For the scavenging assay, 2,2-diphenyl-1-picrylhydrazyl (DPPH) (Sigma-Aldrich, San Louis, MO, USA, 1898-66-4) and (*S*)-Trolox methyl ether (Sigma-Aldrich, Buchs/Zurich, Switzerland, 135806-59-6) were used.

### 2.2. Preparation and Optimization of NP PLA-RSV

The polylactic acid-resveratrol nanoparticles (NP PLA-RSV) were prepared using the emulsion-evaporation technique. Resveratrol and PLA were dissolved in an organic acetone phase, while polyvinyl alcohol (PVA) was dissolved in distilled water to obtain the aqueous phase. The organic phase was added dropwise to the aqueous phase under constant stirring to form an oil-in-water emulsion. The emulsion was then agitated to facilitate solvent evaporation and nanoparticle [7]. For particle size and zeta potential, dynamic light scattering (DLS) and electrophoretic light scattering (ELS) were performed, respectively, using a Nanosizer NANO ZS90 (Malvern Panalytical, Malvern, UK). All measurements were made at room temperature and under standard equipment conditions.

To quantify encapsulated resveratrol, the nanoparticles were subjected to ultracentrifugation at 130,000× *g* in a ultracentrifuge XL-100 (Beckman Coulter, Brea, CA, USA), and the supernatant containing the unencapsulated Resveratrol was separated [8]. The concentration of free resveratrol was determined by UV-Vis spectrophotometry at 305 nm. The encapsulation efficiency was calculated by subtracting the amount of unencapsulated Resveratrol from the total amount, as follows:

$$Encapsulation\;efficiency\;\left(\%\right)=\frac{RSV\;total-RSV\;free}{RSV\;total}\times 100$$

### 2.3. DPPH Scavenging Assay

The antioxidant capacity of optimized NP PLA-RSV was evaluated using the 2,2-diphenyl-1-picrylhydrazyl (DPPH) scavenging assay, following the method described by Echagaray et al. with some modifications [9]. A DPPH solution was prepared at 0.1 mM and protected from light. Then, 5 different concentrations of the nanoparticles were prepared, each mixed with an equal volume of DPPH, and incubated at room temperature in the dark for 10 min.

Subsequently, absorbance was measured at 517 nm using the non-sampled DPPH solution as a methanol target and negative control, and Trolox (0.6 mM) as a positive control. The entrapment capacity was calculated through the following equation.

$$DPPH\;scavenging\;\left(\%\right)=\frac{A_{control}-A_{sample}}{A_{control}}\times 100$$

### 2.4. Hemolysis Assay

The hemocompatibility of the nanoparticles was assessed by a hemolysis assay using human erythrocytes from a male person. Peripheral blood was collected in tubes containing EDTA anticoagulant, centrifuged at 1500 rpm for 10 min, and the erythrocytes were separated. The plasma and leukocyte layers were discarded, and the sample was washed three times with physiological saline solution, then centrifuged under the same conditions. Subsequently, the erythrocytes were resuspended in saline or added to the empty and charged nanoparticle systems and incubated for 1 h at 37 °C. As a negative control, physiological saline solution was used, and as a positive control, 1% Triton was used. After incubation, the samples were centrifuged at 1000× *g* for 10 min, and the supernatant was recovered. The released hemoglobin was quantified by measuring absorbance at 540 nm.

### 2.5. Cell Culture and Experimental Design of the in Vitro Assay

Breast cancer cell lines MCF-7 and MDA-MB-231 were used to perform the cytotoxicity test. The cells were maintained in DMEM medium supplemented with 12% bovine fetal serum and 1% antibiotics (Pen/Strep). Once the cells reached confluence, they were transferred to 96-well plates at a concentration of 5 × 10^4^ cells per well and incubated for 24 h at 37 °C with 95% relative humidity and 5% CO_2_.

Doxorubicin (5 µM) was used as a positive control. For the combined experiments, cells were first exposed to doxorubicin for 12 h, after which the medium was replaced with fresh medium, and the cells were exposed to PLA-RSV nanoparticles for 2 h. Viability was then determined using the crystal violet technique. The two-hour exposure time was selected to assess the acute effects of the nanoparticles following doxorubicin pretreatment and to evaluate their influence on the initial cytotoxic response.

#### Cytotoxicity Assay

Cytotoxicity of the nanoparticles was evaluated using the crystal violet assay, which allows determining cell viability as a function of cell density [10]. At the end of the experiments, the cells were placed in a solution of 0.5% crystal violet with 20% methanol and stirred for 20 min; the excess crystal violet was then removed, and the cells were left to dry at room temperature. The dye retained by the cells was solubilized using 10% acetic acid, stirred for 20 min, and the absorbance was measured at 570 nm.

### 2.6. Statistical Analysis

For the development and optimization of PLA nanoparticles loaded with RSV, a Plackett—Burman design of experiments was initially carried out (initially 4 variables at 2 levels), giving 16 experimental runs; later, a response surface experimental design of only two factors at three levels to run 9 experiments was carried out to optimize the formulation, and the analysis was conducted in StatGraphics 19.

For cytotoxicity analysis and biomarker measurement, the OriginLab 2025 program was used, along with a one-way ANOVA followed by multiple comparisons of means using Fisher’s test with *p* < 0.05. Data are expressed as mean ± SEM from three independent experiments performed in triplicate (*n* = 3).

## 3. Results

### 3.1. Development, Optimization, and Characterization of PLA-RSV Nanoparticles

To develop and optimize PLA nanoparticles with Resveratrol, an experimental design was used in two stages. Initially, a Plackett-Burman screening design was used to evaluate the effects of four formulation and process variables at two levels, resulting in 16 experimental runs. The factors evaluated included the amounts of PLA and Resveratrol, the PVA concentration, the volumes of the organic solvent and aqueous phase, and the stirring time.

Based on the statistical analysis, PLA and RSV amounts were identified as the most significant factors influencing the nanoparticles’ characteristics, whereas the remaining variables did not show statistically significant influence over the dependent variables. (*p* < 0.05); therefore, their previously standardized values were retained (Table 1).

**Figure 1:**
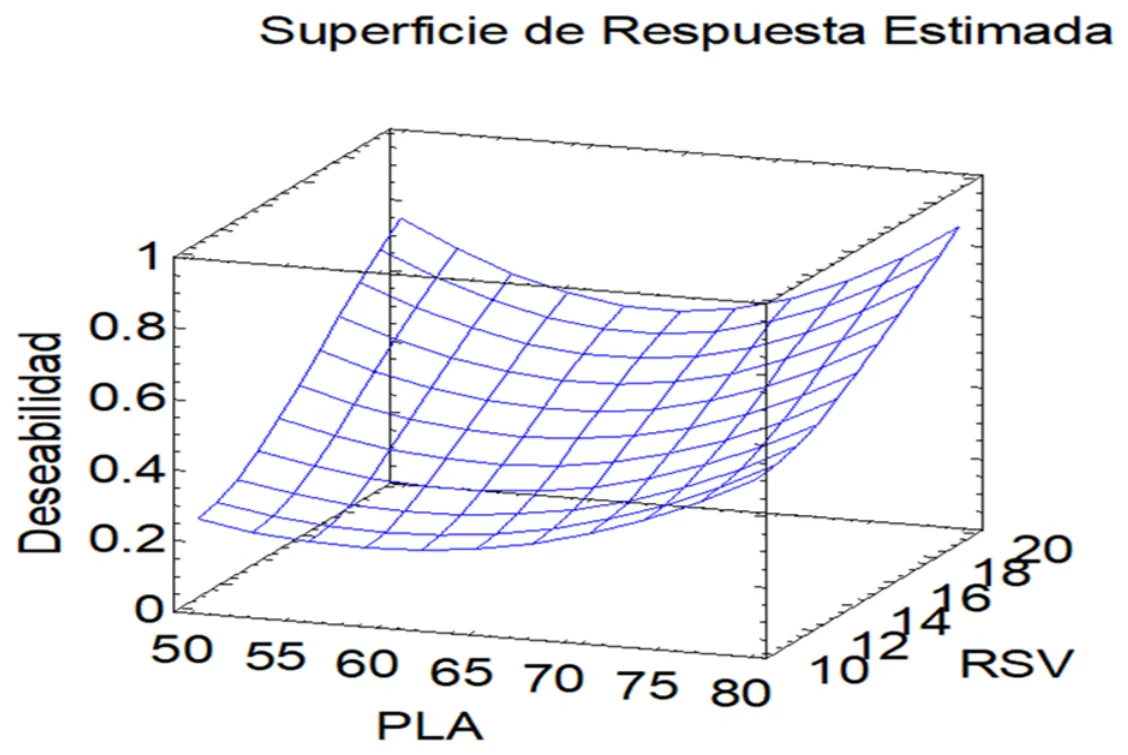
3-dimensional “desirability” graph for optimization.

Subsequently, a surface response analysis was conducted to optimize the formulation across nine systems (Figure 1). The independent variables (X) were the amounts of PLA and RSV, while the dependent variables (Y) were particle size and encapsulation efficiency (Table 2).

**Figure 2:**
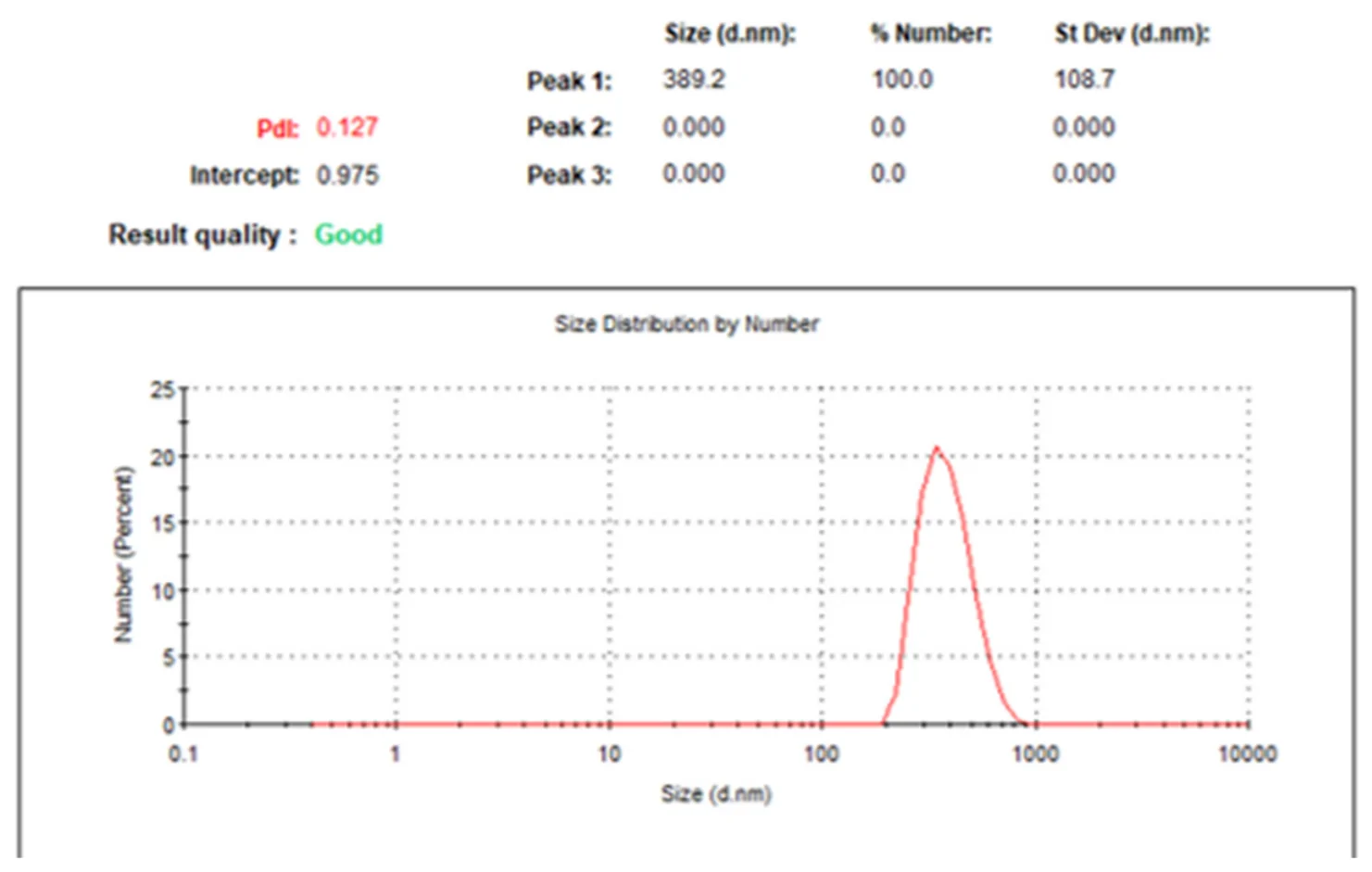
Optimized nanoparticle particle size distribution.

**Figure 3:**
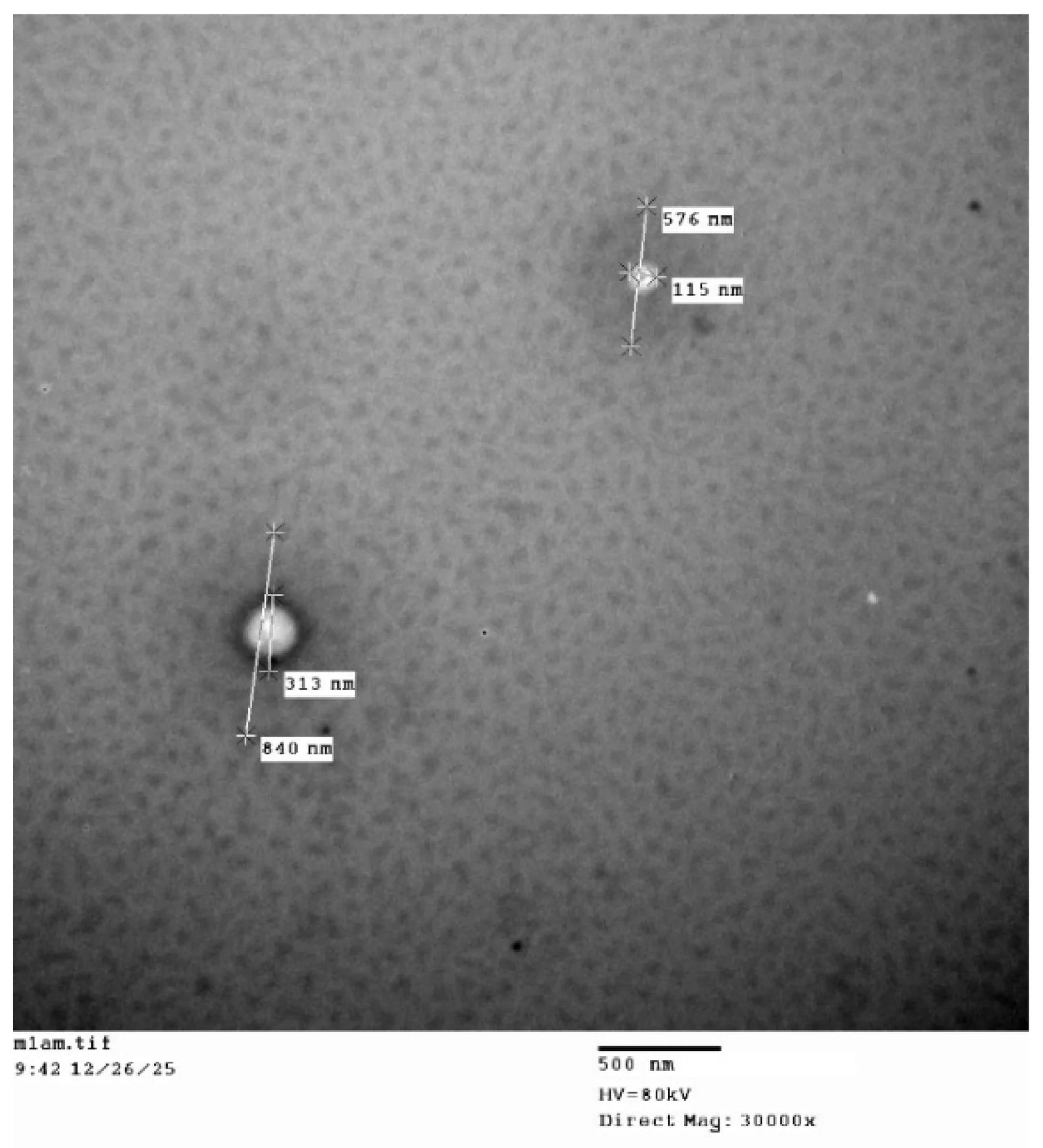
Transmission electron microscopy of the optimized system.

Based on this analysis, system 8 was identified as the optimal system (Table 3). Therefore, transmission electron microscopy was performed on this system to evaluate particle size (Figure 2 and Figure 3), as well as DPPH scavenging, hemolysis, and cytotoxicity assays in cells.

### 3.2. DPPH Scavenging Results

**Figure 4:**
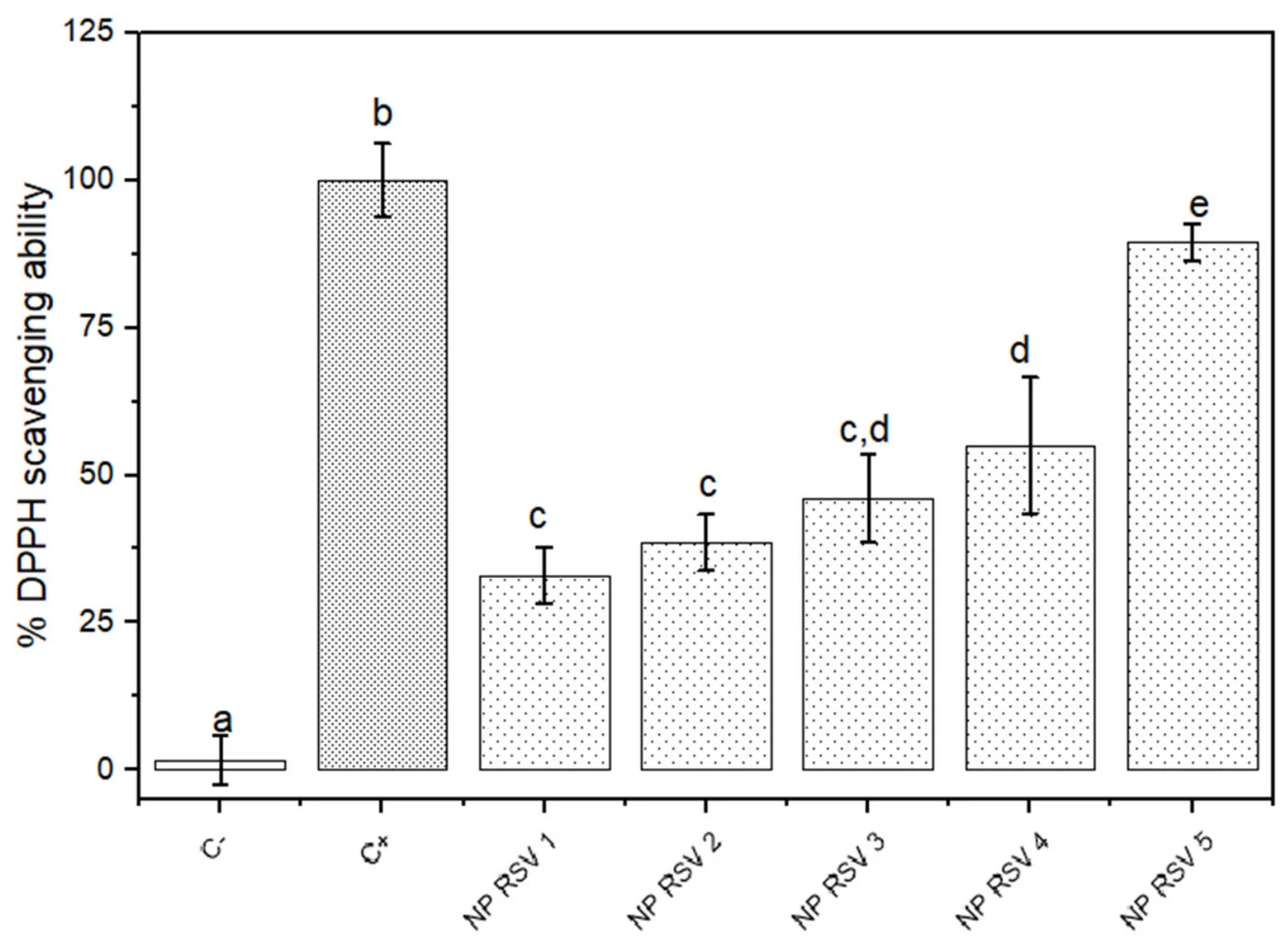
Results of the DPPH scavenging ability of different concentrations of the optimized system. NP RSV 1 (0.0025 mg/mL), NP RSV 2 (0.005 mg/mL), NP RSV 3 (0.01 mg/mL), NP RSV 4 (0.02 mg/mL), NP RSV 5 (0.04 mg/mL), C+ (Trolox 0.6 mM). Bars with different letters indicate significant differences between the means (*p* < 0.05). Data are expressed as mean ± SEM from three independent experiments performed in triplicate (*n* = 3).

NP PLA-RSV was able to eliminate the DPPH free radical, with the effect concentration-dependent (Figure 4). From concentrations equal to or greater than 0.0025 mg/mL, NP PLA-RSV significantly decreased the presence of the free radical in the negative control (*p* < 0.05). In addition, the increase reached approximately 90% at a concentration of 0.04 mg/mL. This behavior suggests that encapsulated resveratrol preserves its antioxidant activity and that this effect is dose-dependent.

### 3.3. Hemolysis Results

**Figure 5:**
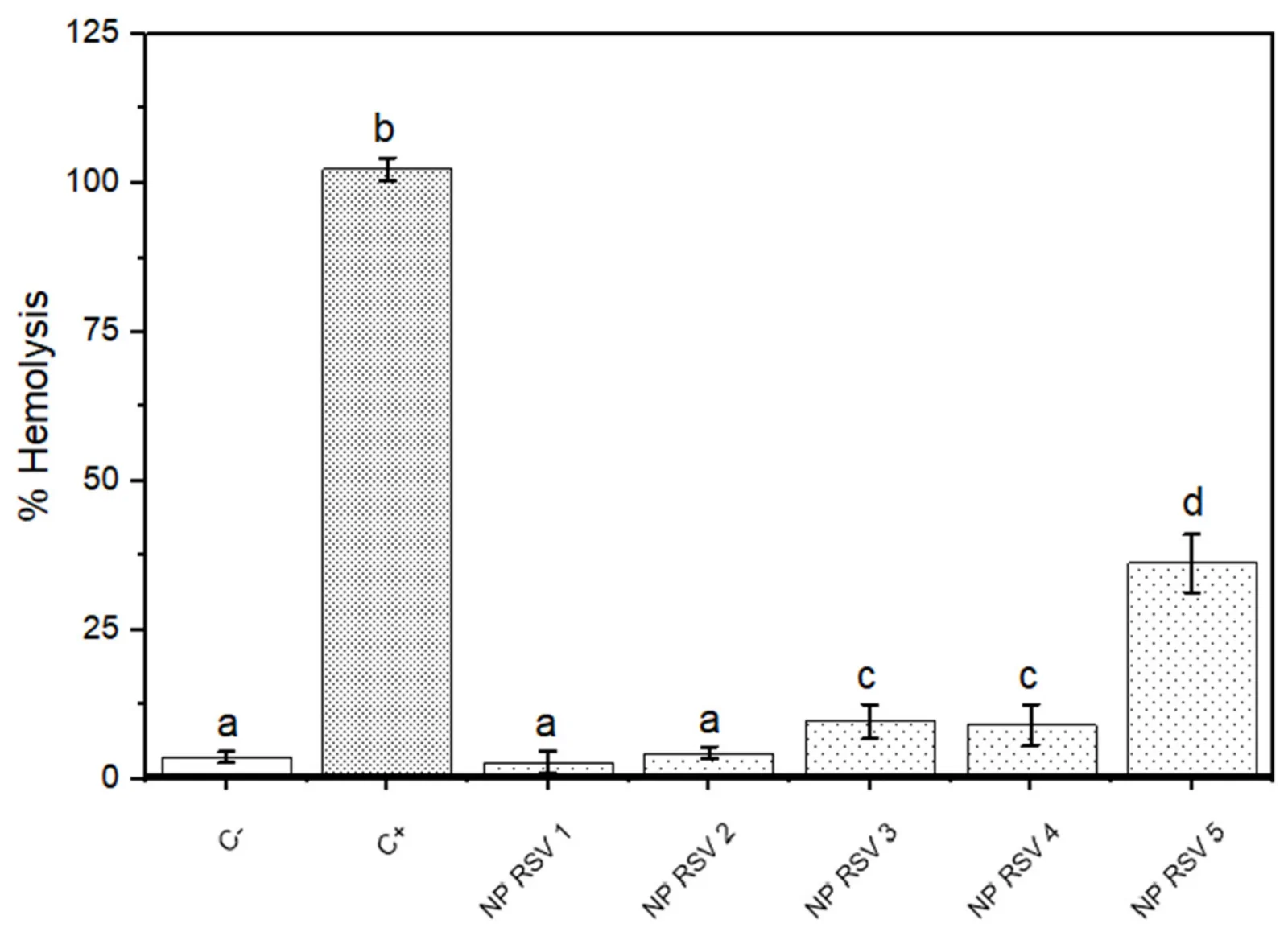
Results of the hemolysis assay of different concentrations of the optimized system. NP RSV 1 (0.0025 mg/mL), NP RSV 2 (0.005 mg/mL), NP RSV 3 (0.01 mg/mL), NP RSV 4 (0.02 mg/mL), NP RSV 5 (0.04 mg/mL), C+ (Doxorubicin 5 µM). Bars with different letters indicate significant differences between the means (*p* < 0.05). Data are expressed as mean ± SEM from three independent experiments performed in triplicate (*n* = 3).

NP PLA-RSV at the first two concentrations showed no significant difference in hemolysis compared with the negative control, indicating good compatibility with erythrocytes (Figure 5). In contrast, a concentration of 0.04 mg/mL significantly increased the percentage of hemolysis compared with the negative control (*p* < 0.05) but remained below that of the positive control, suggesting a dose-dependent effect.

### 3.4. Cytotoxicity Results

**Figure 6:**
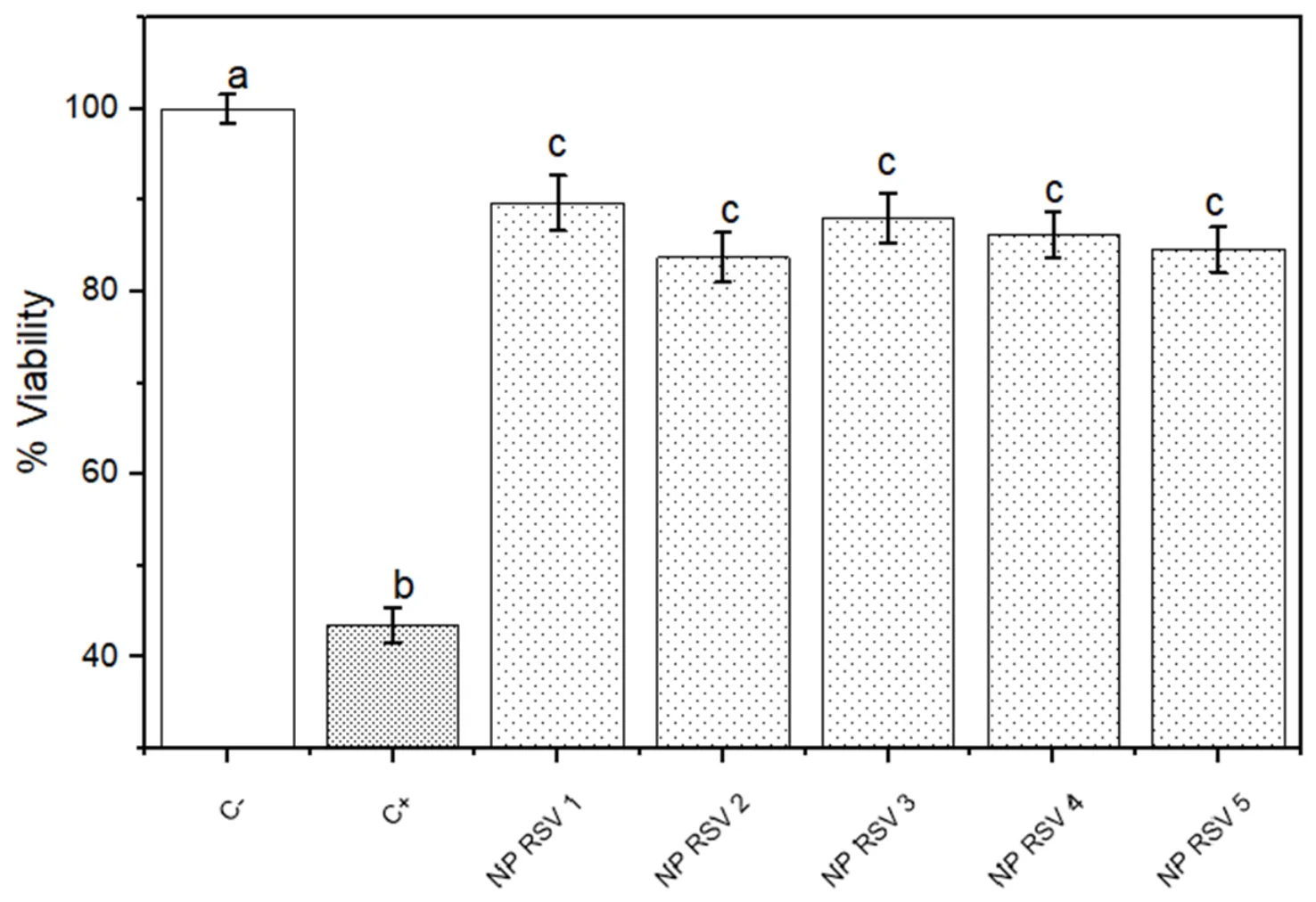
Crystal violet of MCF-7 cells exposed to NP RSV. NP RSV 1 (0.0025 mg/mL), NP RSV 2 (0.005 mg/mL), NP RSV 3 (0.01 mg/mL), NP RSV 4 (0.02 mg/mL), NP RSV 5 (0.04 mg/mL), C+ (Doxorubicin 5 µM). Bars with different letters indicate significant differences between the means (*p* < 0.05). Data are expressed as mean ± SEM from three independent experiments performed in triplicate (*n* = 3).

**Figure 7:**
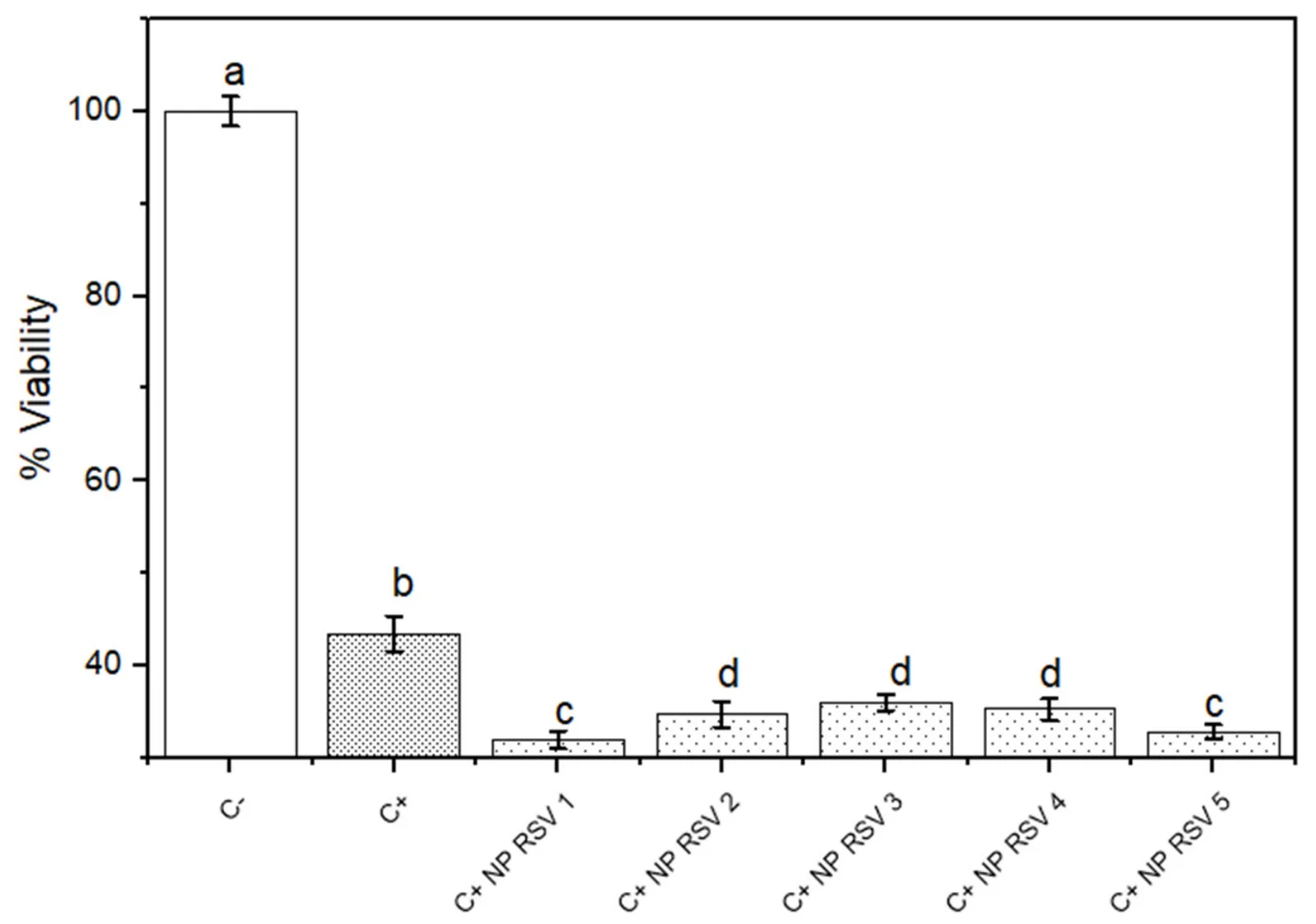
Crystal Violet of MCF-7 cells exposed to NP RSV and Doxorubicin. NP RSV 1 (0.0025 mg/mL), NP RSV 2 (0.005 mg/mL), NP RSV 3 (0.01 mg/mL), NP RSV 4 (0.02 mg/mL), NP RSV 5 (0.04 mg/mL), C+ (Doxorubicin 5 µM). Bars with different letters indicate significant differences between the means (*p* < 0.05). Data are expressed as mean ± SEM from three independent experiments performed in triplicate (*n* = 3).

Exposure of MCF-7 cells to NP PLA-RSV at the different concentrations used significantly decreases cell viability by 15–20% compared with the negative control (Figure 6). The statistical analysis did not show a dose-effect relationship during exposure of MCF-7 cells to PLA-RSV2 NPs. However, the observed effect was lower than that of the positive control (doxorubicin treatment), indicating that NP PLA-RSV alone induces a lower effect on cell viability in MCF-7 cells. In contrast, MCF-7 cells pre-exposed to doxorubicin and then to NP PLA-RSV showed significantly reduced cell viability, exceeding that observed in the positive control (Figure 7). This effect indicates enhanced cytotoxicity when NPs are administered with doxorubicin, with greater cytotoxicity in combination treatments than in individual treatments, on the cytotoxic response to the chemotherapeutic agent.

**Figure 8:**
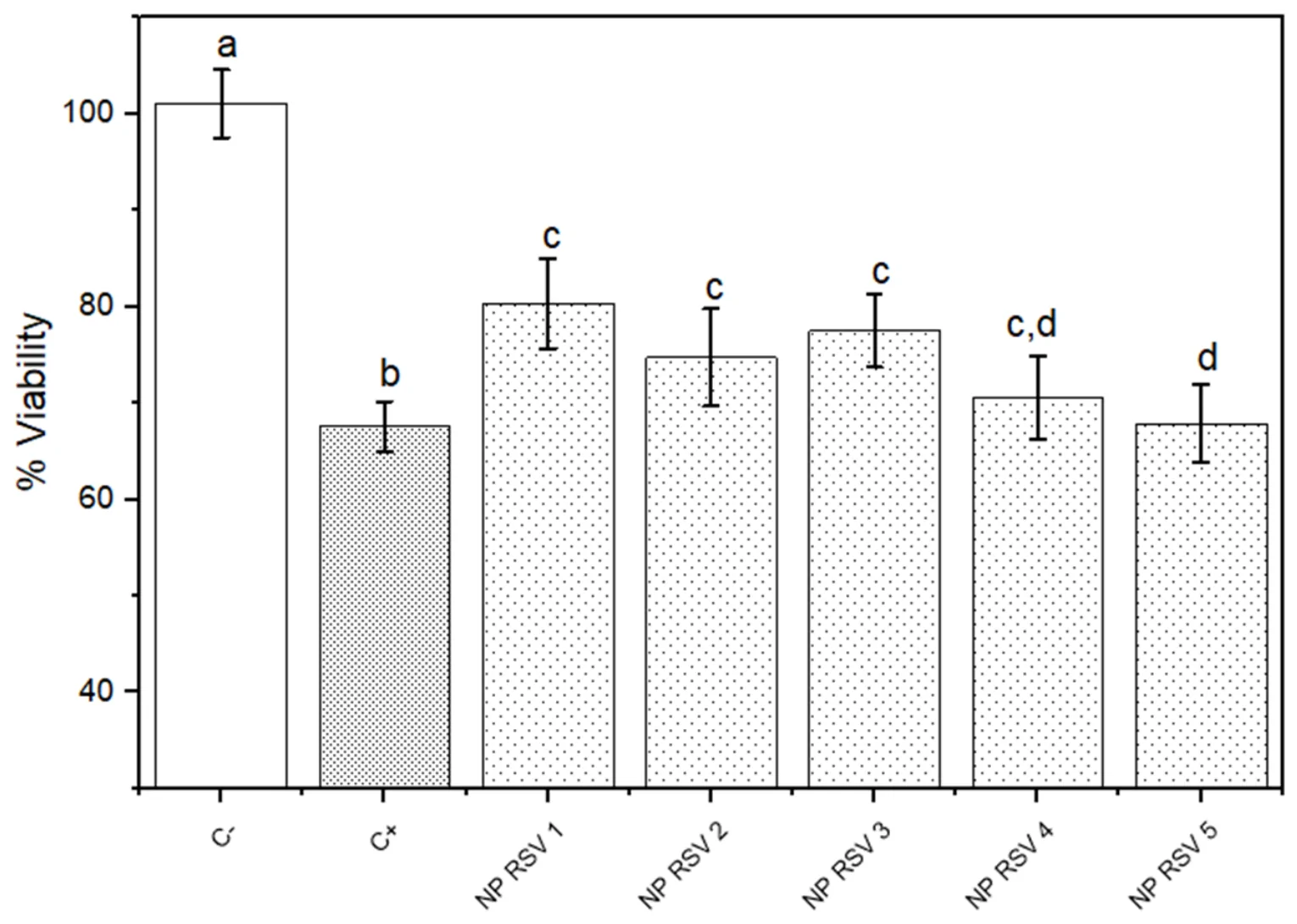
Crystal violet of MDA-MB-231 cells exposed to NP RSV. NP RSV 1 (0.0025 mg/mL), NP RSV 2 (0.005 mg/mL), NP RSV 3 (0.01 mg/mL), NP RSV 4 (0.02 mg/mL), NP RSV 5 (0.04 mg/mL), C+ (Doxorubicin 5 µM). Bars with different letters indicate significant differences between the means (*p* < 0.05). Data are expressed as mean ± SEM from three independent experiments performed in triplicate (*n* = 3).

MDA-MB-231 cells exposed to NP PLA-RSV showed a significant decrease in cell viability compared with the negative control; however, it was lower than that observed in the positive control (Figure 8). The reduction in viability was approximately 20–30%, compared with MCF-7 cells, which showed greater toxicity.

**Figure 9:**
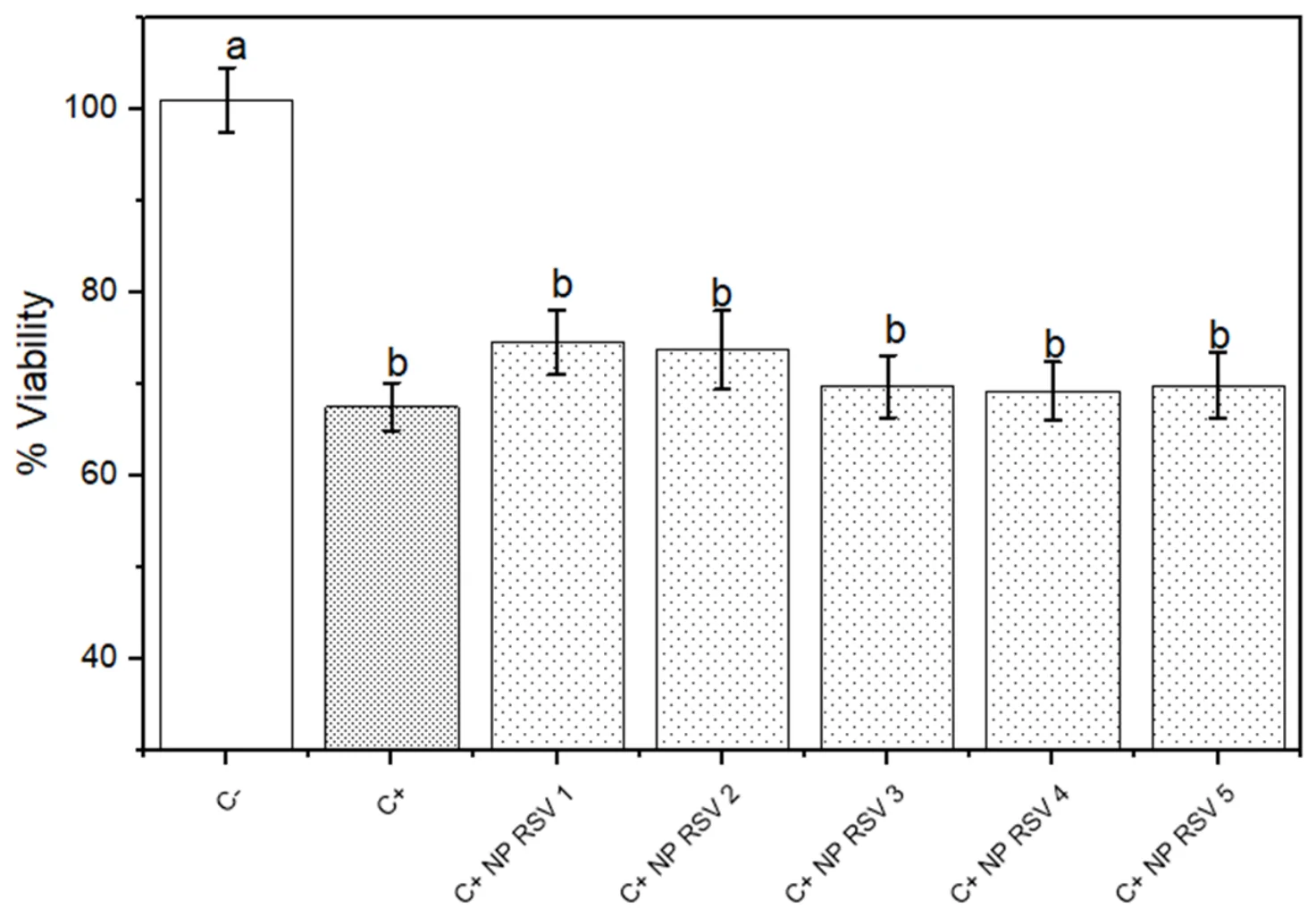
Crystal violet staining of MDA-MB-231 cells exposed to NP RSV and Doxorubicin. NP RSV 1 (0.0025 mg/mL), NP RSV 2 (0.005 mg/mL), NP RSV 3 (0.01 mg/mL), NP RSV 4 (0.02 mg/mL), NP RSV 5 (0.04 mg/mL), C+ (Doxorubicin 5 µM). Bars with different letters indicate significant differences between the means (*p* < 0.05). Data are expressed as mean ± SEM from three independent experiments performed in triplicate (*n* = 3).

MCF-7 cells showed that the combination of doxorubicin and NP PLA-RSV significantly reduced cell viability, whereas MDA-MB 231 cells did not show an additional decrease. No reduction in cell viability was observed in the combined treatments compared to the individual treatments in MDA-MB-231 cells (Figure 9). These results suggest a cell-type-dependent differential response, with MCF-7 cells more sensitive to doxorubicin combination therapy, whereas MDA-MB-231 cells are more sensitive to NP PLA-RSV alone.

## 4. Discussion

During optimization of the NP PLA-RSV, it was found that the PLA: RSV ratio was the primary factor governing the system’s physicochemical properties. Some reports suggest that increasing the polymer content increases particle size and decreases polydispersity, whereas increasing the active ingredient content may favor molecular packaging and improve loading efficiency [11,12]. In contrast, variations in solvent volume, stirring time, and surfactant concentration did not produce significant changes in physicochemical properties under the conditions evaluated. This may be due to the method of preparation, since operational variables such as time and solvent volume tend to have less impact on this type of method [13].

This behavior is consistent with recent articles highlighting that polymer: drug stoichiometry is more closely associated with physicochemical properties and is therefore a critical factor in nanoparticle formation [5]. It was found that increasing the PLA proportion improved retention of the active ingredient within the polymeric matrix, thereby establishing a more stable colloidal system [2]. Taken together, these results suggest that the PLA: RSV ratio is a key parameter for modulating the size, homogeneity, and encapsulation efficiency of nanoparticles.

The optimized system showed sizes close to 393 nm, low PDI values, and negative surface charge, suggesting a homogeneous nanoparticle population; despite the size, the optimized formulation demonstrated significant biological activity in both cell lines, suggesting that the size did not compromise its biological performance in vitro and is compatible with biological environments (Table 3, Figure 2 and Figure 3). Although sizes below 200 nm are usually associated with improved cellular internalization via endocytosis, nanoparticles between 300 and 500 nm have been reported to offer important advantages in tumor models due to their distinct interactions with the extracellular matrix and greater retention in tumor microenvironments [4,5,6,7,8,9,10,11,12,13,14]. On the other hand, the z-potential obtained can help minimize electrostatic interactions with biological membranes and plasma proteins, reducing the formation of a protein corona and activation of the complement system, phenomena associated with positively charged nanoparticles [15,16].

The more than 70% encapsulation efficiency achieved in the optimized system is relevant from a pharmacological perspective, as RSV has low water solubility, rapid metabolism, and limited bioavailability when administered as a non-encapsulated molecule. Therefore, PLA encapsulation is a strategy to mitigate these limitations and improve the pharmaceutical profile [3]. Regarding its antioxidant activity, the DPPH assay demonstrated a dose-dependent response, indicating that RSV retains the ability to neutralize reactive species (Figure 4) [17]. However, several studies have described that the encapsulation of polyphenolic compounds, such as RSV, can acquire a pro-oxidant profile instead of an antioxidant profile in tumor cells, which at the cellular level favors apoptosis mediated by the loss of mitochondrial membrane potential [4,18].

In the hemolysis assay, the optimized system across various concentrations exhibited a low hemolytic index, indicating an acceptable compatibility profile for potential parenteral use. Similar results have been reported with PLGA nanoparticles, which showed low interaction with erythrocyte membranes and no significant hemolysis (Figure 5) [19]. This test is important as it allows us to rule out doses that may generate unwanted effects.

In cytotoxicity assays, NP PLA-RSV showed a dose-dependent decrease in viability in both cell lines (Figure 6 and Figure 9). This behavior suggests intracellular internalization and release of RSV, which coincides with studies showing that resveratrol nanoformulations increase their bioavailability and cytotoxic potency in tumor cells [20,21]. On the other hand, MDA-MB-231 cells exhibited greater cytotoxicity upon exposure to NP PLA-RSV. This may be because these cells, as triple-negative cell lines, have more metabolically active pathways, and resveratrol has been reported to decrease proliferation in this cell type by modulating reactive oxygen species, Bax/Bcl-2, and caspase 3, even in the absence of additional chemotherapeutic agents [22].

The greater sensitivity observed in MDA-MB-231 cells may be associated with their triple-negative phenotype, which is characterized by high metabolic activity, while, in oxidative stress, there is a greater dependence on the regulation of the redox-regulated pathways and intrinsic resistance mechanisms, which have been identified as determinants of the differential response to nanoparticulate systems [23]. On the other hand, the combination with doxorubicin increased cytotoxicity in MCF-7 cells compared to individual treatments, which may be associated with the fact that resveratrol can act as a tumor-sensitizing agent, by modulating the reactive oxygen species pathway, and inhibiting survival pathways, as well as reducing drug resistance through the MDR1/P-gp pathway which facilitates the intracellular accumulation of antineoplastic drugs such as doxorubicin [24,25,26]. In addition, it has been reported that Resveratrol can interfere with transcription factors involved in the stress response, such as NF-kB and Nrf2, thereby promoting apoptosis and improving the efficacy of antineoplastic agents [27]. In subsequent studies, it will be important to elucidate the specific cellular and molecular mechanisms related to the redox modulation promoted by the resveratrol nanoparticles we studied. These studies should focus on understanding the effects on cell proliferation and death through the use of caspase and necroptosis inhibitors, which appear to be useful in the crystal violet assay [28,29], or overexpression or knockdown assays to determine the nature of cell death [30].

The physicochemical characteristics of the optimized PLA-RSV nanoparticles could be associated with the biological effects observed in the present study. The high encapsulation efficiency suggests that resveratrol is incorporated into the cell. Low PDI values and a homogeneous population could favor interactions with the cell. These characteristics, together with the retained antioxidant activity of the encapsulated compound, could contribute to the cytotoxic effects observed in both cell lines and in combination with doxorubicin; therefore, physicochemical properties seem to influence both nanoparticle formation and the observed biological response.

## 5. Conclusions

From a broader perspective, the present study demonstrated that the nanoencapsulation of resveratrol in polymeric nanoparticles is an effective strategy to overcome the physicochemical limitations inherent to resveratrol, such as its low solubility and bioavailability. On the other hand, system optimization revealed that the PLA: RSV ratio is a critical parameter for modulating nanoparticle size, encapsulation efficiency, and zeta potential, which may influence their interactions with biological systems. The optimized system demonstrated adequate blood compatibility and retained its antioxidant capacity, suggesting that encapsulation does not compromise its biological activity. In addition, at the biological level, NP PLA-RSV induced dose-dependent cytotoxicity in the two breast cancer models studied, demonstrating a differential response associated with the metabolic characteristics and redox status of each cell line.

Among the cell lines, MDA-MB-231 cells showed greater sensitivity to nanoparticle treatment, whereas MCF-7 cells showed greater sensitivity to doxorubicin in combination, supporting the potential of resveratrol as a tumor-sensitizing agent. These findings suggest that NP PLA-RSV not only acts as an efficient delivery system but can also modulate cellular events associated with redox state, cell viability, and response to chemotherapeutic drugs. Therefore, the development of this system offers a promising platform for future research to elucidate the mechanisms involved, including biomarkers of stress, apoptosis, and drug resistance. Overall, this work evidenced the therapeutic potential of PLA-RSV NPs both in unique therapy and in combined schemes, strengthening their projection as an alternative approach to breast cancer.

## Figures and Tables

**Table 1:** First design of experiments (Table shows average values).

RSV (mg)	PVA (mg)	Vol PVA (mL)	Stirring Time (min)	Particle Size (nm)	PDI	Zeta Potencial (mV)	Encapsulation Efficiency (%)
10	1	60	10	438.9	0.103	−6.1	40.1
5	1	60	20	250.1	0.164	−4.1	16.4
5	1	30	20	412	0.212	−3.7	51.9
5	3	60	10	383	0.123	−4.3	32.1
7.5	2	45	15	183	0.111	−8.8	35.1
10	3	30	20	371	0.187	−21.2	47.9
10	1	30	10	242	0.121	−11.8	54.8
10	1	60	20	568	0.256	−5.0	39.2
5	3	30	10	228	0.167	−2.6	6.5
5	1	30	10	179	0.245	−10.7	39.9
7.5	2	45	15	400	0.197	−2.6	18.2
10	3	30	20	216.8	0.147	−0.8	37.9
5	3	60	20	492	0.243	−0.8	23.8
7.5	2	45	15	483.6	0.264	−3.8	4.3
10	3	60	10	414	0.177	−1.2	5.7

**Table 2:** Experimental design and composition of PLA nanoparticles with resveratrol (Table shows average values).

System	PLA (mg)	RSV (mg)	Encapsulation Efficiency (%)	Particle Size (nm)	PDI
1	65.0	15.0	43.0	319.8	0.175
2	65.0	10.0	12.0	394.1	0.173
3	65.0	20.0	62.0	115.3	0.273
4	50.0	20.0	54.6	402.3	0.131
5	50.0	15.0	37.8	477.4	0.191
6	80.0	10.0	67.9	409.6	0.163
7	50.0	10.0	21.7	422.9	0.06
8	80.0	20.0	76.0	389.2	0.127
9	80.0	15.0	49.0	416.8	0.103

**Table 3:** Physicochemical characterization of optimized system.

NPs	Particle Size (nm)	Z Potencial (mV)	PDI	Encapsulation Efficiency (%)
**NP RSV**	389.2 +/− 108.7	−2.5	0.127	76.0

## Data Availability

The raw data supporting the conclusions of this article will be made available by the authors on request.

## Abbreviations

PLA	Polylactic acid
RSV	Resveratrol
NPs	Nanoparticles
DPPH	2,2-difenil-1-picrilhidrazil
DLS	Dynamic light scattering
PVA	Polyvinyl alcohol
